# Association Between Glycemic Control and Complications With Concentration of Urinary Exfoliated Proximal Tubule Kidney Cells in People With Diabetes Mellitus

**DOI:** 10.1155/jdr/1273073

**Published:** 2025-01-16

**Authors:** Henry H. L. Wu, Venkatesha Bhagavath, Long The Nguyen, Rajkumar Chinnadurai, Ewa M. Goldys, Carol A. Pollock, Sonia Saad

**Affiliations:** ^1^Renal Research Laboratory, Kolling Institute of Medical Research, Royal North Shore Hospital & The University of Sydney, Sydney, Australia; ^2^ARC Centre of Excellence for Nanoscale Biophotonics, School of Biomedical Engineering, The University of New South Wales, Sydney, Australia; ^3^Biostatistics Support and Consultation Services, Northern Sydney Local Health District, Sydney, Australia; ^4^Faculty of Biology, Medicine and Health, The University of Manchester, Manchester, UK; ^5^Department of Renal Medicine, Royal North Shore Hospital, Northern Sydney Local Health District, Sydney, Australia

**Keywords:** cell exfoliation, diabetes mellitus, diabetic kidney disease, proximal tubule cells, retinopathy, urine

## Abstract

**Background:** Emerging evidence suggests cell exfoliation could be operating under the control of cell metabolism. It is unclear if there are associations between the concentration of exfoliated kidney proximal tubule cells (PTCs) in urine with glycemic control and complications. Our study is aimed at exploring this.

**Methods:** Urine samples were collected from 122 adult study participants and stored at −80°C. Exfoliated PTCs were extracted from thawed urine using a validated specific immunomagnetic separation method based on anti-CD13 and anti-SGLT-2 antibodies. The number of PTCs was assessed using brightfield microscopy. Study participants were grouped into those with no diabetes mellitus (DM) and those with DM. Individuals with DM were further subgrouped into those with and without retinopathy. Adjusted Poisson regression analysis was conducted for the DM cohort, investigating associations between demographic, clinical, and biochemical parameters with mean urinary exfoliated PTCs.

**Results:** The adjusted Poisson regression analysis noted sex to have a significant association with mean number of urinary exfoliated PTCs, with a lower incidence rate in males compared to females (incidence rate ratio (IRR) 0.56, 95% CI 0.35–0.89, *p* = 0.014). Each 1% increase in glycated haemoglobin (HbA1c) was associated with an increase of 1.03 times in mean exfoliated PTCs (IRR 1.03, 95% CI 1.01–1.04, *p* = 0.007), and DM patients with retinopathy had an increase of 1.68 times in mean exfoliated PTCs compared to those without retinopathy (IRR 1.68, 95% CI 1.07–2.62, *p* = 0.024). No significant associations were observed with albuminuria or estimated glomerular filtration rate (eGFR).

**Conclusions:** Our results indicate increased shedding of PTCs into the urinary tract in patients with poorer glycemic control, particularly those with diabetic retinopathy and in females.

## 1. Introduction

Cell exfoliation is an active biochemical process that has been linked to the homeostasis of mammalian organs. Exfoliation occurs when extracellular matrix components that usually tightly connect between cells within a structure break off, with external cells being removed from the epithelial luminal surface [1]. Activity of cell exfoliation is thought to be under the control of cell metabolism and is now being deemed a potentially useful indicator of metabolic status [2]. In particular, kidney cell exfoliation into urine has been previously found to have significant links with diabetes mellitus (DM), reflecting metabolic dysregulation [3, 4]. Proximal tubule cell (PTC) exfoliation could be especially relevant in DM given glucose entry into PTCs is insulin-dependent, making PTCs especially sensitive to hyperglycemia in hyperglycemic conditions [5]. Numerous studies have demonstrated increased shedding of podocytes and extracellular vesicles into urine with poor metabolic control and glomerulosclerosis development in DM [6–8]. However, there are no studies to date that evaluated associations between the number of exfoliated PTCs and glycemic control, alongside related macro and microvascular complications of DM such as cardiovascular disease, diabetic kidney disease (DKD), and in particular retinopathy, which is the most common microvascular complication of DM and typically one of the early complications to manifest in patients with advanced diabetic disease [9]. Our study is aimed at exploring this.

## 2. Methods

### 2.1. Study Participant Recruitment and Ethical Considerations

The inclusion criteria for study participant recruitment are adult individuals of either sex aged between 18 and 75 years of age under the care of Royal North Shore Hospital and/or North Shore Private Hospital, Sydney, Australia, with and without Type 1 or 2 DM, and with serum samples obtained within 72 h before urine sample collection. Individuals aged < 18 years or > 75 years were excluded from the study. As per the current Australian Diabetes Society guidelines and for the purposes of this study in grouping study participants into “cases” (i.e., people with DM) and “controls” (i.e., people without DM), DM status is defined by individuals presenting with serum glycated haemoglobin (HbA1c) ≥ 6.5%, fasting glucose ≥ 7.0 mmol/L, random glucose ≥ 11.1 mmol/L, and those on a 75-g oral glucose tolerance test displaying fasting glucose ≥ 7.0 mmol/L or 2 − h glucose ≥ 11.1 mmol/L [10]. Study recruitment was conducted prospectively in which participants were only recruited upon their presentation to the departmental clinic, with no prior knowledge nor planning on recruitment of specific individuals. Informed consent was obtained from all study participants. Data collection in this study was carried out in accordance with relevant local guidelines and regulations, and the collection of human data was approved by the human ethics committee at Royal North Shore Hospital and North Shore Private Hospital (Ref: HREC/17/HAWKE/471) and the University of New South Wales, Sydney, Australia (Ref: HC180710).

A total of 122 study participants were recruited. There were 38 individuals with no DM and 84 individuals with DM, of which there were 26 individuals with DM and retinopathy and 58 individuals with DM but without retinopathy.

### 2.2. Extraction and Quantification of Urinary Exfoliated Proximal Tubule Kidney Cells

**Figure 1:**
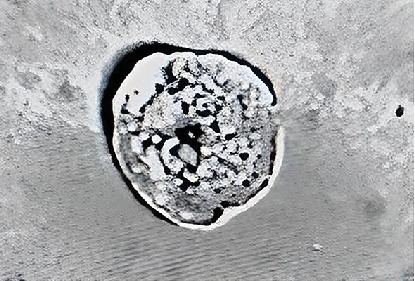
Example of a urinary exfoliated proximal tubule kidney cell specifically extracted via a validated specific immunomagnetic separation method based on anti-CD13 and anti-SGLT2 antibodies.

Using urine bottles with a capacity of up to 100 mL, spot urine samples were collected from adult individuals who fulfilled the above criteria and the volume of urine collected (milliliters) was recorded by H.H.L.W. for each study participant. Each collected urine sample was placed on ice immediately after collection for transportation to the Renal Research Laboratory, Kolling Institute of Medical Research, and was centrifuged for 20 min at 4°C to collect exfoliated cells and then washed twice with phosphate-buffered saline (PBS). Prior to extraction of PTCs, the initially processed samples were to be stored at −80°C with PBS and 10% dimethyl sulfoxide (10% DMSO) [11]. Whilst urine may contain a wide range of different cells—podocytes, proximal and distal tubular cells, stem/progenitor cells, extracellular vesicles, epithelial cells, bladder, and collecting duct—it has been previously established that CD13 and sodium–glucose linked transporter-2 (SGLT-2) antibodies are uniquely expressed in PTCs [11]. Specific PTCs expressing CD13, SGLT-2, and angiotensinogen were extracted via a validated specific immunomagnetic separation method based on anti-CD13 and anti-SGLT2 antibodies that our group has previously described (Figure 1) [11] and then quantified in brightfield microscopy.

### 2.3. Collecting and Summarizing Demographic, Clinical, and Biochemistry Data

Each study participant's demographic information including their age (years), sex and ethnicity, and clinical data encompassing medical history and diagnoses such as the presence of cardiovascular disease and retinopathy was collected from the New South Wales Health Powerchart electronic database. Height (meters) and weight (kilograms) were measured and recorded on the day of urine sample collection, and data in relation to body mass index (BMI) was automatically generated. Biochemistry data recorded for the purposes of this study included HbA1c (%), estimated glomerular filtration rate (eGFR) (mL/min/1.73 m^2^) by the Chronic Kidney Disease Epidemiology Collaboration Equation [12], urinary albumin-to-creatinine ratio (uACR), and serum creatinine (*μ*mol/L). HbA1c, eGFR, and serum creatinine data were obtained from serum tests collected within 72 h of urine sample collection. uACR data was obtained from another urine sample collected on the same day which was sent to New South Wales Health Pathology for analysis. Systolic and diastolic blood pressure (BP) were recorded on the same day using an automated Omron Hem 7142T1 BP Monitor with different cuff sizes available depending on an individual's suitability. The estimated creatinine clearance (milliliters per minute) for each study participant was generated from MDCalc through inputting relevant information into the Cockcroft–Gault equation [13], as it informs us specifically on tubular creatinine clearance [14].

Patient demographics alongside clinical and biochemical data were summarized using appropriate descriptive statistics. Given variables are nonnormally distributed or ordinal, the median and interquartile range (IQR) were used to summarize continuous variables. Proportions with percentages were presented for categorical variables. When comparing between the no DM versus DM groups (i.e., two comparator groups), continuous variables were compared using the Mann–Whitney *U* test, and categorical variables were compared using the chi-square test or Fisher's exact test accounting for sparsely distributed data. When comparing between the DM with retinopathy versus the DM without retinopathy versus the no DM group (i.e., three comparator groups), continuous variables were compared using the Kruskal–Wallis *H* test, and categorical variables were compared using the chi-square test or Fisher's exact test accounting for sparsely distributed data.

### 2.4. Conducting Poisson Regression Analysis

A Poisson regression analysis model was used to determine the significance of the association between diabetic status, other study variables, and the number of urinary exfoliated PTCs. A Poisson regression is a quantitative research methodology specifically used to analyse count data as the dependent variable [15]. The Poisson regression model is the simplest count regression model [15]. Coefficients are exponentiated, since counts must be 0 or greater. A Poisson regression assumes a Poisson distribution, which is often characterized by a substantial positive skew with most cases falling at the low end of the dependent variable's distribution, and a variance that equals the mean. Because count data distributions (i.e., number of urinary exfoliated PTCs) will have a Poisson distribution, a Poisson regression tends to fit these data better than linear regression does (which assumes a normal distribution) [15]. As a result, predictive relationships with a dependent variable (i.e., number of urinary exfoliated PTCs) can be examined as in ordinary linear regression, but without the problems from having the nonnormal distributions that are expected with count data [15].

Adjusted Poisson regression analysis was conducted in this study. The model investigated associations between each included variable of the study and the number of urinary exfoliated PTCs for the DM cohort, adjusting for other covariates within the statistical model. The incidence rate ratio (IRR) along with their corresponding 95% CI and *p* value were calculated to determine the significance of association for each variable and mean urinary exfoliated PTCs. The model incorporated HbA1c, eGFR, BMI, age, sex, ethnicity, urine volume, uACR, serum creatinine, estimated creatinine clearance, systolic and diastolic BP, history of cardiovascular event(s), and incidence of retinopathy as covariates. Statistical analysis was performed with *p* < 0.05 being the level of significance. Analysis was performed in SPSS (Version 26).

## 3. Results

In our cohort of 122 study participants, the diabetic group was significantly older compared to the nondiabetic group (63.8 vs. 51.5 years, *p* < 0.001). Median BMI was higher in the diabetic compared to the nondiabetic group (25.6 vs. 23.2, *p* = 0.005). Median HbA1c was higher in the diabetic compared to the nondiabetic group (7.4 vs. 6.1%, *p* = 0.016). Considering all study participants, the range between the highest and lowest recorded HbA1c is 8.6%. Median serum creatinine levels were higher in the diabetic compared to the nondiabetic group (115.5 vs. 96.0 *μ*mol/L, *p* = 0.028), reflecting a lower median eGFR in the diabetic compared to the nondiabetic group (52 vs. 66 mL/min/1.73 m^2^, *p* = 0.042). The proportion of patients with macroalbuminuria was higher in the diabetic compared to the nondiabetic group (36.8 vs. 17.9%, *p* = 0.044). The proportion of patients with a history of cardiovascular event(s) was more prevalent in the diabetic compared to the nondiabetic group (53.6 vs. 18.4%, *p* < 0.001). Diabetic retinopathy was observed in 32% of individuals with DM. Median urinary exfoliated PTCs were higher in the diabetic compared to the nondiabetic group (6 vs. 5, *p* = 0.021). A summary of demographic, clinical, and biochemistry parameters for the diabetic and nondiabetic groups is presented in Table 1.

Subdividing the study participants within the diabetic group into those with DM and retinopathy and those with DM but without retinopathy, whilst also considering those in the nondiabetic group, individuals with DM and retinopathy were older compared to diabetic individuals without retinopathy and individuals without DM (67.5 vs. 66.5 vs. 50.5 years, *p* < 0.001). BMI was higher in individuals with DM and retinopathy compared to individuals with DM but without retinopathy and individuals without DM (27.1 vs. 25.5 vs. 23.2, *p* = 0.017). Median HbA1c was higher in the group with DM and retinopathy compared to those with DM but without retinopathy and those without DM (7.6 vs. 7.3 vs. 6.1%, *p* < 0.001). The proportion of patients with a history of a cardiovascular event(s) was more prevalent in the group with DM and retinopathy compared to those without retinopathy (73.1 vs. 44.8 vs. 18.4%, *p* < 0.001). Median urinary exfoliated PTCs were higher in those with DM and retinopathy compared to those with DM but without retinopathy and those without DM (8 vs. 6 vs 5, *p* = 0.002). A summary of demographic, clinical, and biochemistry parameters for study participants with DM and retinopathy, study participants with DM but without retinopathy, and the nondiabetic group is presented in Table 2.

In order to evaluate associations between a number of urinary exfoliated PTCs, glycemic control, and other covariates, a Poisson regression analysis was conducted for the 84 study participants diagnosed with DM (Table 3). Sex was found to display a significant association with mean number of exfoliated PTCs, with a lower incidence rate for males compared to females (IRR 0.56, 95% CI 0.35–0.89, *p* = 0.014), and significantly lower number of exfoliated PTCs in males (mean 5.58, 95% CI 4.74–6.43) compared to females (mean 10.00, 95% CI 5.83–14.17). HbA1c displayed significant associations with the number of exfoliated PTCs, where each 1% increase in HbA1c is associated with an increase of 1.03 times of mean exfoliated PTCs (IRR 1.03, 95% CI 1.01–1.04, *p* = 0.007). Individuals diagnosed with retinopathy exhibited increased numbers of exfoliated PTCs (IRR 1.68, 95% CI 1.07–2.62, *p* = 0.024), but other variables such as eGFR and serum albumin:creatinine ratio did not demonstrate statistically significant associations with a number of urinary exfoliated PTCs (*p* > 0.05).

## 4. Discussion

Overall, our results indicate increased shedding of PTCs into the urinary tract in patients with DM compared to those without DM, in those who are female compared to males, in patients with progressively poorer glycemic control, and in patients with DM and retinopathy compared to those without retinopathy.

The relationship between kidney cell exfoliation, glycemic control, and associated diabetic complications has not been explored explicitly in the past. In our study, we have demonstrated a significant increase in the shedding of PTCs into the urinary tract in individuals with progressively poorer glycemic control and patients with DM and retinopathy compared to those without retinopathy. As microvascular endothelial cells are specific targets of intracellular hyperglycemia-induced injury, multiorgan microvascular endothelial cell dysfunction is likely to increase with elevated HbA1c [16–19]. This process is mainly attributed to four hallmark mechanisms—namely, protein kinase C activation, activation of the hexosamine pathway, activation of the polyol pathways, and subsequently the formation of advanced glycation end (AGE) products [20, 21]. Although it is not yet proven, it is likely that worsening endothelial cell dysfunction alongside increased cellular inflammation, oxidative stress, and apoptotic processes eventually leads to local vascular structural changes, where increased activity of cell exfoliation occurs as part of dysregulated autophagy [2, 22]. Such changes at a larger, systemic scale in organs embedded with microvasculature such as the kidneys may occur as microvascular disease progresses with DM. The intercellular junctions and desmosomes which connect between different cell types within a structure may gradually loosen and break off leading to increased exfoliation of cells into the extracellular spaces [1]. This could be the pathway in which kidney cell exfoliation is likely affected by the severity of endothelial cell dysfunction with systematically worsened microvascular disease in DM [23]. This mechanism may explain for the increased number of exfoliated PTCs into urine with poorer glycemic control as observed from our analysis. It is also plausible that links between the number of exfoliated PTCs into urine and retinopathy, the most common microvascular complication of DM, are explained by this cascade of pathophysiological events. Further studies are required to determine the causality of the associations observed in our study.

Otherwise, previous investigation, comparing the ability to grow urinary exfoliated kidney cells in culture between people with DM and its associated macro and microvascular complications versus healthy and diabetic individuals free of complications, has been conducted. A seminal study by Detrisac et al. [3] found few cultivable exfoliated cells in healthy adults and diabetic patients free of complications, but significantly greater amounts of cultivable exfoliated kidney cells in individuals with diabetic retinopathy and/or advanced DKD. Nevertheless, Detrisac et al. [3] did not quantitate the cells in that study and did not specifically assess PTCs. Considering our data displayed more exfoliated PTCs in individuals with elevated HbA1c, it is not surprising that more exfoliated cells in the advanced diabetic group grew in culture previously.

Whilst there are reasonable physiological postulations to explain for the associations between a number of urinary exfoliated PTCs, elevated HbA1c, and retinopathy, it is surprising that such significant associations between a number of urinary exfoliated PTCs and kidney function in the Poisson regression analysis were not observed. This is despite serum creatinine and the proportion of study participants with macroalbuminuria being significantly increased and eGFR being significantly decreased in the diabetic group compared to the nondiabetic group. PTCs make up the majority of the kidney mass, and PTC histopathology is a hallmark representation of multiple kidney diseases (including DKD) and correlates with kidney function decline [24, 25]. Previous animal and translational studies have established increased shedding of podocytes and extracellular vesicles into urine with poor glycemic control and glomerulosclerosis development in DM [6, 8, 26]. In particular, an increased number of exfoliated podocyte microparticles across the DKD spectrum is observed, with this being shown to prognosticate DKD progression [7, 8, 27]. Numerous exosomal enzymes found in exfoliated kidney cells, such as glycogen synthase kinase-3 beta (GSK-3*β*) which is mostly detected in higher levels in podocytes but also in PTCs with DM and DKD severity, have been shown to display greater prognostic accuracy for DKD progression when compared with albuminuria [4, 28]. Multiple studies noted exosomal WT-1 from urine correlates with the severity of proteinuria, the extent of glomerular damage, and the rate of kidney function decline in patients with DM [29, 30]. Considering our findings, the exact mechanisms that explain for the associations between the activity of PTC exfoliation into urine and DKD progression may be more complex than previously thought, and further study is needed.

There are no published studies at present that investigated the role of sex differences in affecting the number of urinary exfoliated PTCs. Our Poisson regression analysis results demonstrated increased shedding of PTCs into the urinary tract in females compared to males. Whilst our analysis may potentially reflect the sex-associated links to urinary PTC exfoliation in both nondiabetic and diabetic individuals, a more demographically and ethnically diverse sample is required to validate this early hypothesis. Furthermore, additional studies should be conducted to address whether the number of urinary exfoliated PTCs is affected by different therapies including conventional Western medicine, as well as herbal medicines and natural products used by many patients for treatment of DM [31–34].

Reflecting on the methodology of our study in garnering these results, there are strengths of our study methodology to acknowledge. We managed to perform a validated long-term cryopreservation protocol and reliably demonstrated noninvasive isolation of assessable exfoliated PTCs from 122 human urine samples using immunomagnetic extraction with CD13, SGLT-2 antibodies, and magnetic beads applied in consistent concentrations. The reliability and reproducibility of this protocol are important steps to allow for reliable identification and quantification of urinary exfoliated PTCs in individuals with varying degrees of glycemic control to fulfill the main aim of our study. The Poisson regression model considered many relevant covariates in its analysis outside of glycemic control, including renal function parameters such as uACR and serum creatinine, and relevant comorbidities such as a history of cardiovascular event(s). Based on current understanding, each of the included demographic, clinical, and biochemistry parameters could play a role in affecting cellular metabolism and hence the activity of PTC exfoliation into urine.

There are limitations to our study approach in quantifying PTC concentration in urine. It is likely that poor glycemic control increases diuresis, and hence, the absolute increase in the number of exfoliated PTCs may well be underestimated by our approach. Adjusting for the concentration of urine volume depends on an individual's water intake, and this is challenging to objectively control for given human factors. It would be similarly difficult to determine the concentration of different cell types and molecules that may be exfoliated in urine alongside PTCs. Another consideration relates to the collection of spot urine samples versus a “timed” urine sample collected across different timepoints. Diurnal variation of metabolic activity may impact on the degree of PTC exfoliation which may influence results. This is challenging to control in practice considering the multitude of human and practical factors which can affect the reliability of urine samples collected over different timepoints. These limitations should be subject to further investigation going forward.

In summary, our findings provide a novel observation that suggests the presence of significant associations between the number of exfoliated PTCs into urine with progressively poorer glycemic control and diabetic retinopathy. Whilst a convincing association with DKD is yet to be demonstrated, additional research is required to explore the pathophysiological mechanisms that may underpin an intricate relationship that exists between the activity of PTC exfoliation into urine and metabolic changes that occur in diabetes.

## Figures and Tables

**Table 1:** Demographic, clinical, and biochemistry characteristics of the study cohort based on diabetes mellitus status (*n* = 122).

**Variable**	**Total (** **n** = 122**)**	**No DM (** **n** = 38**)**	**DM (** **n** = 84**)**	**p** ** value** ^**a**^
Age (years), mean (SD)	60.0 (14.3)	51.5 (16.2)	63.8 (11.6)	< 0.001
Sex, *n* (%)				
Female	36 (29.5)	20 (52.6)	16 (19.1)	< 0.001
Male	86 (70.5)	18 (47.4)	68 (81)	
Ethnicity, *n* (%)				
0 (White)	68 (55.7)	23 (60.5)	45 (53.6)	0.474
1 (non-White)	54 (44.3)	15 (39.5)	39 (46.4)	
Height (m), median (IQR)	1.69 (0.10)	1.71 (0.19)	1.69 (0.13)	0.439
Weight (kg), median (IQR)	76.2 (18.4)	68.8 (18.3)	72.4 (25.1)	0.011
BMI, median (IQR)	26.4 (5.2)	23.2 (6.1)	25.6 (8.6)	0.005
HbA1c (%), median (IQR)	7.3 (3.2)	6.1 (3.3)	7.4 (3.2)	0.016
eGFR (mL/min/1.73 m^2^), median (IQR)	60.4 (32.4)	66 (50)	52 (56.5)	0.042
Urine volume (mL), median (IQR)	71 (15)	77 (48)	68 (65)	0.170
uACR, median (IQR)	23.1 (65.6)	12.8 (90)	80.6 (158.7)	0.068
Serum creatinine (*μ*mol/L), median (IQR)	107.5 (89)	96 (60)	115.5 (106)	0.028
Urinary exfoliated proximal tubule cells, median (IQR)	6 (5)	5 (7)	6 (6)	0.021
Systolic BP (mmHg), median (IQR)	133.5 (33)	128 (26)	136.5 (34.5)	0.393
Diastolic BP (mmHg), median (IQR)	75 (15)	74.5 (21)	75 (14)	0.353
Albuminuria, *n* (%)				
Macro	29 (23.8)	14 (36.8)	15 (17.9)	0.044
Micro	50 (41.0)	13 (34.2)	37 (44.1)	
History of CV events, *n* (%)				
No	70 (57.4)	31 (81.6)	39 (46.4)	< 0.001
Yes	52 (42.6)	7 (18.4)	45 (53.6)	
Retinopathy, *n* (%)				
No	95 (77.9)	38 (100)	57 (67.9)	< 0.001
Yes	27 (22.1)	0 (0)	27 (32.1)	

Abbreviations: BMI, body mass index; BP, blood pressure; CV, cardiovascular; DM, diabetes mellitus; eGFR, estimated glomerular filtration rate; HbA1c, glycated haemoglobin; IQR, interquartile range; SD, standard deviation; uACR, urine albumin-to-creatinine ratio.

^a^Continuous variables were compared between the diabetic and nondiabetic groups using the Mann–Whitney *U* test. Categorical variables were compared using the chi-square test or Fisher's exact test accounting for sparsely distributed data.

**Table 2:** Demographic, clinical, and biochemistry characteristics of the study cohort in which study participants with diabetes mellitus are grouped based on retinopathy status (*n* = 122).

**Variable**	**No DM (** **n** = 38**)**	**DM without retinopathy (** **n** = 58**)**	**DM with retinopathy (** **n** = 26**)**	**p** ** value** ^**a**^
Age (years), median (IQR)	50.5 (29)	67.5 (14)	66.5 (24)	< 0.001
Sex, *n* (%)				
Female	20 (52.6)	13 (22.4)	3 (11.5)	< 0.001
Male	18 (47.4)	45 (77.6)	23 (88.5)	
Ethnicity, *n* (%)				
0 (White)	23 (60.5)	30 (51.7)	15 (57.7)	0.680
1 (non-White)	15 (39.5)	28 (48.3)	11 (42.3)	
Height (m), median (IQR)	1.71 (0.19)	1.69 (0.14)	1.70 (0.12)	0.719
Weight (kg), median (IQR)	68.8 (18.3)	71.4 (25.7)	75.4 (24.7)	0.025
BMI, median (IQR)	23.2 (6.1)	25.5 (9.2)	27.1 (8.5)	0.017
HbA1c (%), median (IQR)	6.1 (3.3)	7.3 (1.1)	7.6 (1.2)	< 0.001
eGFR (mL/min/1.73 m^2^), median (IQR)	66 (50)	57 (60)	51.8 (48)	0.110
Urine volume (mL), median (IQR)	77 (48)	60 (41)	62 (47)	0.192
uACR, median (IQR)	12.8 (90)	8.2 (83.5)	18.7 (94.6)	0.068
Serum creatinine (*μ*mol/L), median (IQR)	96 (60)	111 (111)	125 (107)	0.088
Urinary exfoliated proximal tubule cells, median (IQR)	5 (7)	6 (6)	8 (5)	0.002
Systolic BP (mmHg), median (IQR)	128 (26)	132 (40)	139 (21)	0.372
Diastolic BP (mmHg), median (IQR)	74.5 (21)	73 (13)	77 (20)	0.188
Albuminuria, *n* (%)				
Macro	14 (36.8)	10 (17.2)	5 (19.2)	0.248
Micro	13 (34.2)	25 (43.1)	12 (46.2)	
History of CV events, *n* (%)				
No	31 (81.6)	32 (55.2)	7 (26.9)	< 0.001
Yes	7 (18.4)	26 (44.8)	19 (73.1)	

Abbreviations: BMI, body mass index; BP, blood pressure; CV, cardiovascular; DM, diabetes mellitus; eGFR, estimated glomerular filtration rate; HbA1c, glycated haemoglobin; IQR, interquartile range; uACR, urine albumin-to-creatinine ratio.

^a^Continuous variables were compared between the three groups using the Kruskal–Wallis *H* test. Categorical variables were compared using the chi-square test or Fisher's exact test accounting for sparsely distributed data.

**Table 3:** Poisson regression model evaluating the association between covariates and the number of urinary exfoliated proximal tubule cells in study participants with diabetes mellitus (*n* = 84).

**Variable**	**Poisson regression model**	**p** ** value**
	**IRR (95% CI)**	
HbA1c per 1% increase	1.03 (1.01–1.04)	0.007
eGFR per 1 mL/min/1.73 m^2^ increase	1.01 (0.99–1.03)	0.156
BMI per 1 unit increase	1.04 (0.99–1.09)	0.171
Age per 1 year increase	1.01 (0.99–1.03)	0.230
Sex		
Female	0^a^	
Male	0.56 (0.35–0.89)	0.014
Ethnicity		
0 (White)	0^a^	
1 (non-White)	1.25 (0.85–1.86)	0.261
Estimated creatinine clearance per 1 mL/min increase	0.30 (0.09–1.06)	0.061
Urine volume per 1 mL increase	1.001 (1.00–1.01)	0.354
uACR per 1 unit increase	1.001 (1.00–1.003)	0.100
Serum creatinine per 1 *μ*mol/L increase	1.00 (0.99–1.001)	0.827
Systolic BP per 1 mmHg increase	0.99 (0.98–1.01)	0.848
Diastolic BP per 1 mmHg increase	0.99 (0.98–1.01)	0.272
History of CV event		
No	0^a^	
Yes	0.80 (0.54–1.18)	0.263
Retinopathy		
No	0^a^	
Yes	1.68 (1.07–2.62)	0.024

Abbreviations: BMI, body mass index; BP, blood pressure; CV, cardiovascular; eGFR, estimated glomerular filtration rate; HbA1c, glycated haemoglobin; IRR, incidence rate ratio (i.e., ratio of mean urinary exfoliated proximal tubule cells); uACR, urine albumin-to-creatinine ratio.

^a^Reference category used in the estimation of the IRR in the Poisson regression model.

## Data Availability

The data that support the findings of this study are available from the corresponding author upon reasonable request.
